# TeachOpenCADD: a teaching platform for computer-aided drug design using open source packages and data

**DOI:** 10.1186/s13321-019-0351-x

**Published:** 2019-04-08

**Authors:** Dominique Sydow, Andrea Morger, Maximilian Driller, Andrea Volkamer

**Affiliations:** In Silico Toxicology, Institute of Physiology, Charité – Universitätsmedizin Berlin, Charitéplatz 1, 10117 Berlin, Germany

**Keywords:** Computer-aided drug design, Python, RDKit, Open source, Teaching, Learning, Cheminformatics, Structural bioinformatics

## Abstract

Owing to the increase in freely available software and data for cheminformatics and structural bioinformatics, research for computer-aided drug design (CADD) is more and more built on modular, reproducible, and easy-to-share pipelines. While documentation for such tools is available, there are only a few freely accessible examples that teach the underlying concepts focused on CADD, especially addressing users new to the field. Here, we present TeachOpenCADD, a teaching platform developed by students for students, using open source compound and protein data as well as basic and CADD-related Python packages. We provide interactive Jupyter notebooks for central CADD topics, integrating theoretical background and practical code. TeachOpenCADD is freely available on GitHub: https://github.com/volkamerlab/TeachOpenCADD.
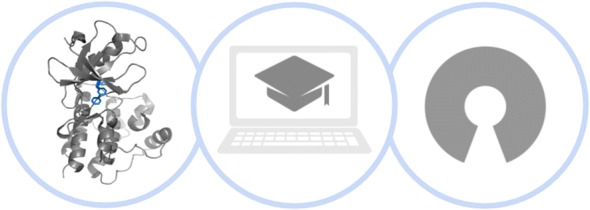

## Introduction

Open access resources for cheminformatics and structural bioinformatics as well as public platforms for code deposition such as GitHub are increasingly used in research. This combination facilitates and promotes the generation of modular, reproducible, and easy-to-share pipelines for computer-aided drug design (CADD). Comprehensive lists of open resources are reviewed by Pirhadi et al. [1], or presented in the form of the web-based search tool Click2Drug [2], aiming to cover the full CADD pipeline.

While documentation for open access resources is available, freely accessible teaching platforms for concepts and applications in CADD are rare. Available examples include the following: On the one hand, graphical user interface (GUI) based tutorials teach CADD basics, such as the web-based educational Drug Design Workshop [3, 4]. On the other hand, examples for educational coding tutorials are the Java-based Chemistry Development Kit (CDK) [5–9] and the Teach–Discover–Treat (TDT) initiative [10], which launched challenges to develop tutorials, such as a Python-based virtual screening (VS) workflow to identify malaria drugs [11, 12].

Complementing these resources, we developed the TeachOpenCADD platform to provide students and researchers new to CADD and/or programming with step-by-step tutorials suitable for self-study training as well as classroom lessons, covering both ligand- and structure-based approaches. TeachOpenCADD is a novel teaching platform developed by students for students, using open source data and Python packages to tackle various common tasks in cheminformatics and structural bioinformatics. Interactive Jupyter notebooks [13] are presented for central topics, integrating detailed theoretical background and well-documented practical code. Topics build upon one another in the form of a pipeline, which is illustrated at the example of the epidermal growth factor receptor (EGFR) kinase, but can easily be adapted to other query proteins. TeachOpenCADD is publicly available on GitHub and open to contributions from the community: https://github.com/volkamerlab/TeachOpenCADD (current release: 10.5281/zenodo.2600909).

## Methods

**Fig. 1:**
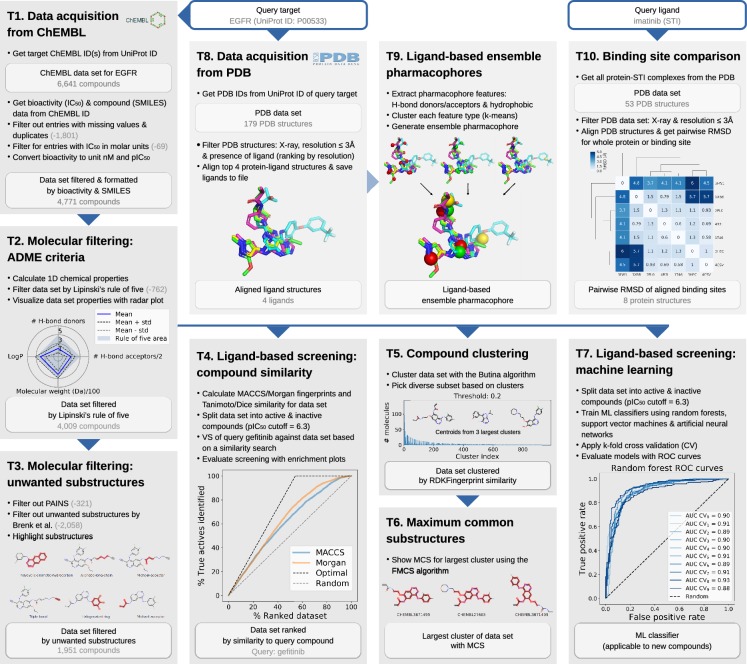
TeachOpenCADD talktorial pipeline. TeachOpenCADD is a teaching platform for open source data and packages, currently offering ten talktorials in the form of Jupyter notebooks on central topics in CADD, ranging from cheminformatics (T1–7) to structural bioinformatics (T8–10). The talktorials are illustrated at the example of EGFR (based on data sets from ChEMBL and PDB queries in November 2018)

**Fig. 2:**
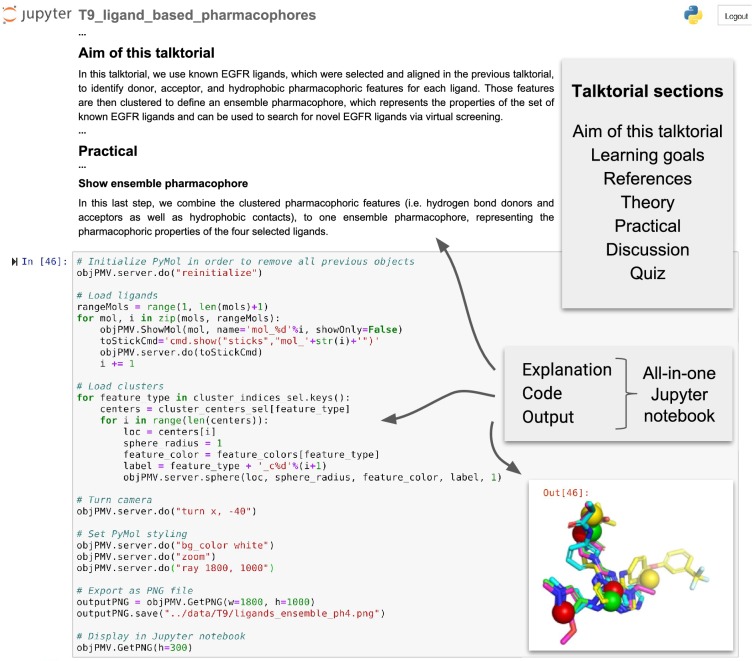
Screenshot of TeachOpenCADD talktorial composition. TeachOpenCADD talktorials are Jupyter notebooks that cover one CADD topic each, composed of (i) a topic motivation, (ii) learning goals, (iii) references to literature, (iv) theoretical background, (v) practical code, (vi) a short discussion, and (vii) a quiz—all in one place. Shown here is a screenshot of parts of talktorial T9 to generate pharmacophores

TeachOpenCADD currently consists of ten *talktorials* covering central topics in CADD, see Fig. 1. Talktorials are offered as interactive Jupyter notebooks that can be used as tutorials but also for oral presentations, e.g. in student CADD seminars (talk + tutorial = talktorial). They start with a topic motivation and learning goals, continue with the main part composed of theoretical background and practical code, and end with a short discussion and quiz, see Fig. 2.

Open data resources employed are the ChEMBL [14] and PDB [15] databases for compound and protein structure data acquisition, respectively. Open source libraries utilized are RDKit [16] (cheminformatics), the ChEMBL webresource client [17] and PyPDB [18] (ChEMBL and PDB application programming interface access), BioPandas [19] (loading and manipulating molecular structures), and PyMOL [20] (structural data visualization). Additionally, basic Python computing libraries employed include numpy [21, 22] and pandas [23, 24] (high-performance data structures and analysis), scikit-learn [25] (machine learning), as well as matplotlib [26] and seaborn [27] (plotting). Furthermore, the user is instructed how to work with conda [28], a widely used package, dependency and environment management tool. A conda yml file is provided to ensure an easy and quick setup of an environment containing all required packages.

The talktorial topics include how to acquire data from ChEMBL (T1), filter compounds for drug-likeness (T2), and identify unwanted substructures (T3). Furthermore, measures for compound similarity are introduced and applied for VS of kinase inhibitor gefitinib (T4) as well as for compound clustering (T5), including the use of maximum common substructures (T6). Machine learning approaches are employed to build models for predicting active compounds (T7). Lastly, protein-ligand complexes are fetched from the PDB (T8), used to generate ligand-based ensemble pharmacophores (T9). Geometry-based binding site comparison of kinase inhibitor imatinib binding proteins is performed to analyse potential off-targets (T10). In summary, the presented talktorials build a pipeline with starting points being (i) a query protein to study associated compound data (T1 and T8) and (ii) a query ligand to investigate associated on- and off-targets (T10), see Fig. 1. These talktorials can be studied independently from each other or as a pipeline.

As an example, the talktorial pipeline is used to identify novel EGFR kinase inhibitors. EGFR kinase is a transmembrane protein, which activates several signaling cascades to convert extracellular signals into cellular responses. Dysfunctional signaling of EGFR is associated with diseases such as cancer, making it a frequent target in drug development projects (the reader is referred to a review by Chen et al. [29] for more information on EGFR). Furthermore, the pipeline can easily be adapted to other examples by simply exchanging the query protein (T1 and T8: protein UniProt ID) and query ligand (T10: ligand names in the PDB).

## Results

In the following, the content of each talktorial is briefly discussed and summarized in Fig. 1. If not noted otherwise, tasks are conducted with RDKit or basic Python libraries as stated in the Methods section. Note that reported numbers and results are based on data sets from ChEMBL and PDB queries conducted in November 2018.

*T1. Data acquisition from ChEMBL.* Compound information on structure, bioactivity and associated targets is organized in databases such as ChEMBL, PubChem [30], or DrugBank [31]. For the query target EGFR (UniProt ID P00533), compound data including molecular structure (SMILES) and bioactivity data is automatically fetched from the ChEMBL database, using the ChEMBL webresource client, and is filtered for e.g. binding assays and IC$_{50}$ measurements (6,641 compounds). The data set is formatted and further filtered: e.g. duplicates and entries with missing values are dropped and only bioactivity values in molar units are kept and converted to pIC$_{50}$ values (4,771 compounds retained, referred to as *data set T1*), see Fig. 1.T1.

*T2. Molecular filtering: ADME criteria.* Not all compounds are suitable starting points for drug development due to undesirable pharmacokinetic properties, which for instance negatively affect a drug’s absorption, distribution, metabolism, and excretion (ADME). Therefore, such compounds are usually not included in data sets for VS. *Data set T1* is filtered by lead-likeness criteria, i.e. Lipinski’s rule of five [32], in order to remove less drug-like molecules from the EGFR data set (4009 compounds retained, referred to as *data set T2*). This data set is visualized using radar plots demonstrating their ADME properties, see Fig. 1.T2, and serves as starting point for several talktorials discussed in the following.

*T3. Molecular filtering: unwanted substructures.* Compounds can contain unwanted substructures that may cause mutagenic, reactive, or other unfavorable pharmacokinetic effects [33] or that may lead to non-specific interactions with assays (PAINS) [34]. Such unwanted substructures are detected and highlighted in *data set T2*. This knowledge can be integrated into cheminformatics pipelines to either perform an additional filtering step before screening (1,951 compounds retained) or – more often – to set alert flags to compounds being potentially problematic. They can be manually evaluated by medicinal chemists if reported as hits after screening, see Fig. 1.T3.

*T4. Ligand-based screening: compound similarity*. In VS, compounds similar to known ligands of a target under investigation often constitute the starting point for drug development. This approach follows the similar property principle stating that structurally similar compounds are more likely to exhibit similar biological activities [35, 36] (exceptions are so-called activity cliffs [37]). For computational representation and processing, compound properties can be encoded in the form of bit arrays, so-called molecular fingerprints, e.g. MACCS [38] and Morgan fingerprints [39, 40]. Compound similarity can be assessed by comparison measures, such as the Tanimoto and Dice similarity [41]. Using these encoding and comparison methods, VS is conducted based on a similarity search: the EGFR inhibitor gefitinib is used to find its most similar compounds in data set T2. With the data being split into active and inactive compounds based on the chosen pIC${_{50}}$ cutoff of 6.3, screening results are evaluated with enrichment plots, see Fig. 1.T4. In the top 5% of the compounds ranked by similarity, called the enrichment factor at 5% (EF$_{5\%}$), 8.3% of actives can be retrieved, while the random and optimal EF$_{5\%}$ of this data set are 5.0% and 9.2%, respectively.

*T5. Compound clustering.* The similar property principle can also be used to identify groups of similar compounds via clustering, in order to pick a set of diverse compounds from these clusters for e.g. non-redundant experimental testing. In this talktorial, Butina clustering [42] based on the RDKFingerprint [43] is applied to cluster *data set T2* at a Tanimoto distance cutoff of 0.2, resulting in 988 clusters with the largest cluster consisting of 143 compounds, see Fig. 1.T5. Following the example in the TDT pipeline by Riniker et al. [11], a maximum of 1000 compounds is subsequently picked by selecting the ten most similar compounds per cluster (or 50% for clusters with fewer compounds), starting with the largest cluster. Thereby, compound diversity is ensured (representatives of each cluster), while structure-activity relationship (SAR) information is retained (most similar compounds selected from clusters).

*T6. Maximum common substructures*. In order to visualize shared scaffolds and thereby emphasize the extent and type of chemical similarities or differences of a compound cluster, the maximum common substructure (MCS) [44] can be calculated and highlighted. The MCS for the largest cluster from T5 is calculated using the FMCS algorithm [45], see Fig. 1.T6. Different parameters can be applied, e.g. a threshold to set the percentage of compounds in the set that need to share the same MCS, or a restriction to match ring bonds only with other ring bonds.

*T7. Ligand-based screening: machine learning.* With the continuously increasing amount of available data, machine learning (ML) gained momentum in drug discovery and especially in ligand-based VS to predict the activity of novel compounds against a target of interest. The EGFR compound data set is split into active and inactive compounds as described in T4, and used to train ML classifiers based on random forests (RF) [46], support vector machines (SVM) [47], and artificial neural networks (ANN) [48], applying 10-fold cross validation. Models are evaluated using receiver operating characteristic (ROC) curves and mean area under the curve (AUC) values (mean AUC results for RF, SVM, and ANN are 90%, 87%, and 87%, respectively), see Fig. 1.T7. The trained models can be used to perform a classification of an unknown screening data set to predict novel potential EGFR inhibitors.

*T8. Data acquisition from PDB*. The PDB database holds 3D structural data and meta information on experimentally resolved proteins. Using PyPDB, all EGFR structures are automatically fetched from the PDB (by UniProt ID) and filtered by ligand-bound structures resolved with X-ray crystallography, retaining four EGFR-ligand structures with good structural resolution. Using the Python integration of the molecular visualization tool PyMOL, those structures are subsequently aligned to each other in 3D. Ligands are extracted, see Fig. 1.T8, and saved to be used in T9 for the generation of a ligand-based ensemble pharmacophore.

*T9. Ligand-based ensemble pharmacophores.* Another approach for ligand-based VS – besides a similarity search (T4) or machine learning classifiers (T7) – are ligand-based (ensemble) pharmacophore models. They describe important steric and physicochemical properties of a ligand (or a set of ligands) to bind a target under investigation. Examples for physicochemical properties are so-called donor, acceptor, and hydrophobic pharmacophoric features present in a molecule [49, 50]. For the EGFR ligands selected and aligned in T8, pharmacophoric features are identified for each ligand and subsequently clustered with k-means clustering [51] in order to define an ensemble pharmacophore, see Fig. 1.T9. Such a pharmacophore represents the properties of the set of known EGFR ligands and can be used to search for novel EGFR ligands via VS, as described in an RDKit pharmacophore tutorial by Stiefl et al. [52].

*T10. Off-target prediction and binding site comparison.* Off-targets are proteins that interact with a drug or (one of) its metabolite(s) without being the designated target, potentially causing unwanted side effects. Off-targets mainly occur because they share similar structural motifs in their binding site with on-targets, and are therefore able to bind similar ligands. Computational off-target prediction using binding site comparison is an established approach in early stages of drug development [53, 54]. In T10, structural similarity is exemplarily accessed using a basic measure, i.e. the geometrical variation between structures by calculating the root mean square deviation (RMSD) between pairs of aligned structures using PyMOL, including either the whole proteins or focusing on their binding sites. Pairwise RMSD comparison of seven protein structures binding imatinib, a small molecule tyrosine kinase inhibitor for cancer treatment, is able to separate tyrosine kinases (on-targets) from quinone reductase (reported off-target [55]), see Fig. 1.T10.

## Conclusion

The presented teaching platform TeachOpenCADD aims at introducing interested students and researchers to the ease and benefit of using open access resources for cheminformatics and structural bioinformatics. Jupyter notebooks (talktorials) offer detailed theoretical background and Python code examples, forming an automated pipeline that saves and reloads results from one topic to another. The pipeline is illustrated using the example of EGFR, but can easily be adapted to other examples by exchanging the input protein and ligand. Beyond their teaching purpose for self-study training and classroom lessons, the talktorials can serve as starting point for users’ project-directed modifications and extensions. TeachOpenCADD intends to expand existing and add new topics continuously, and is open for contributions and ideas from the community.

## Abbreviations

CADD: computer-aided drug design
GUI: graphical user interface
CDK: Chemistry Development Kit
TDT: Teach–Discover–Treat
VS: virtual screening
EGFR: epidermal growth factor receptor
ADME: absorption, distribution, metabolism, excretion
SAR: structure–activity relationship
MCS: maximum common substructure
ML: machine learning
RF: random forest
SVM: support vector machine
ANN: artificial neural network
ROC: receiver operating characteristic
AUC: area under the curve
RMSD: root mean square deviation
EF: enrichment factor
